# Validation of facial attributions in leadership: Trustworthiness and age in Chinese mid-level management

**DOI:** 10.1371/journal.pone.0324508

**Published:** 2025-05-27

**Authors:** Jing Rachel Ma, David Ian Perrett

**Affiliations:** 1 School of Psychology & Neuroscience, University of St. Andrews, St Andrews, Scotland, United Kingdom; 2 School of Business, University of Dundee, Dundee, United Kingdom; National Taiwan University, TAIWAN

## Abstract

The attributions made to faces are well described by two dimensions of apparent trustworthiness (valence or warmth) and apparent competence (dominance and power) (Todorov A, Mende-Siedlecki P, Dotsch R. Curr Opin Neurobiol, 2013, 23, 373–80). This model has been extended to include a third dimension of apparent age and attractiveness (Sutherland CAM, Oldmeadow JA, Santos IM, Towler J, Michael Burt D, Young AW. Cognition, 2013, 127, 105–18). Previous research has tested the association between appearance and leadership attainment for high-level leaders such as elite politicians and chief executive officers of top performing organisations in the US and Western Europe. Here we focus on a Chinese organisational context and explore how facial attributions are associated with appointment at mid-level managerial positions. Participants rated leadership, competence, trustworthiness, attractiveness and age of faces of male employees of a Chinese Real Estate company. Our findings reveal that apparent trustworthiness and age are more critical predictors of leadership attainment than competence or attractiveness in the context of mid-level management in China. The study supports the three-dimensional attribution framework and reaffirms the importance of facial cues in leadership selection across diverse cultural settings.

## Introduction

Competence and trustworthiness (warmth or morality) are the two axes of the two-dimensional social evaluation frameworks [1–4] in interpersonal perceptions. Over 75% of the variance in impressions of others can be accounted for by the framework of the these dimensions [5,6], echoing the valence–dominance model [7] which has emerged as the most prominent framework of how people evaluate faces in social perceptions. This framework maps out facial cues to valence (trustworthiness, warmth) and dominance (power, competence) and provides a model for understanding rapid social judgements from the face. Expansions within this theoretical domain suggest incorporating a third dimension—youth/attractiveness—arguably offering a more comprehensive view of real-life social perceptions [2]. The perception of leadership is undeniably influenced by facial appearance and thus affects leadership emergence in real life [8–10].

Apparent competence is the most studied trait that predicts leadership. Across Western societies, perceived competence is found to predict election success at both national and regional levels [9–15]. These results extend to a younger population [16]. Children’s choices of captains are similar in pattern to adults’ choices of a competent leader, and both predict the actual election voting results [16] suggesting relatively stable preferences of apparent competence in leadership.

This research identifying the association between apparent competence from face and real-world leadership choices has been carried out usually in the West and mainly on high-level leaders such as elite politicians [12,14,17–19] and chief executive officers (CEOs) of top performing organisations [20–27]. Other studies have demonstrated that distinctive facial features are favoured for leadership under different contexts. Strong/powerful/competent/masculine faces are chosen for leadership in the context of external conflict (war) whereas warm/trustworthy/approachable/feminine faces are chosen for leadership in the context of internal conflict within an organisation or country [28–32].

Cultural difference is evident in the preference of the appearance of leaders. Rule et al. [9] were one of the first groups to investigate the face – leader emergence link in non-Western societies. Their study found trait judgements from the face can predict leadership selection in America and Japan. In their study, apparent power (dominance and facial maturity) and warmth (likeability and trustworthiness) from the face were perceived similarly across cultures – in America and Japan. The Western participants relied on perceived power-related traits from the face to predict leadership results, while Eastern participants relied on warmth-related apparent traits to predict leadership. Since then, several studies have investigated facial appearance in relation to election results in East Asia and have replicated the finding of leadership associated with traits other than power in East Asia [13,33–35]. Indeed, researchers using implicit bias detection methods found the evidence that senior managers who are high in power are also perceived as having a higher level of competence and warmth in China [36,37], contradicting the well accepted notion that higher power individuals will be perceived as positive in competence and negative in warmth in China [38,39].

Facial social evaluation framework of power (competence) and valence (trustworthiness) offers a sensitive tool for revealing nuanced cultural and contextual variations in social power stereotypes and leadership dynamics. Jones and colleagues [40] collaborated with labs across world regions trying to determine whether the valence-dominance social evaluation framework [7] is truly universal. Using the same data analytical method - Principal Component Analysis (PCA) with orthogonal components, Jones’s team replicated the finding in most world regions except for Eastern Europe, although they also report finding a third dimension in most of the world regions which Todorov excluded. Jones et al. also proposed an alternative analysis – PCA with non-orthogonal rotation, to allow more dimensions to emerge and found 2–4 main factors underlying facial impressions in different world regions. In Todorov’s original study [41], they indeed found that power (competence) and valence (trustworthiness) account for 80% of the variance in judgements inferred from the face which left room for a third dimension underrepresented. Sutherland et al. [2] argue that attractiveness/youth is a third face dimension of social evaluation (additional to the dimensions of power and trustworthiness). These findings show that power (competence) and valence (trustworthiness) are stable major facial impression factors across cultures while variance exists in different world regions as to whether one or two additional factors, i.e., attractiveness and age, are employed to form social judgements over facial features.

There is an apparent preference for attractiveness in the face of leaders [42–44]; attractive candidates are paid more than average-looking colleagues [45], and are favoured in recruitment [46] and elections [11,15,19]. Being more attractive than average can decrease apparent trustworthiness [47]. Although there is a high correlation between attractiveness and valence (trustworthiness and warmth) [41], the two domains can be differentiated.

Maturity and its opposite, baby-facedness [14,48], are another well studied social trait in leadership success [14,48–53]. For example, leaders with older-looking faces are preferred in traditional knowledge domains, whereas younger-looking leaders are preferred for new challenges [51]. Facial attractiveness and maturity are intertwined attributions. Ageing in adult faces decreases perceived attractiveness but increases perceived power to a certain extent [14,48,52]. The relationship between age and perceived power is, however, non-linear with perceived power increasing until the age of 35 but not thereafter, for male faces [54].

Despite the abundance of research on facial appearance of leaders in political elections [10–12,14,16,55,56], studies examining these perceptions in the context of business leadership, especially at the mid-level and in cultures outside the West, remain scarce. It is possible that different traits are associated with mid-level manager selection and top-level managers or political leaders. Not only do business managers have drastically different tasks compared to political leaders and chief executive officers but also mid-level managers are often appointed based on a series of assessments, which is different from political leaders who gain their position via public voting [57–63].

The distinction between the roles of top-level executives and mid-level managers further complicates the landscape of leadership perception [64,65]. While the former usually situate the top of organisational hierarchies, and lead via visionary goals and extensive managerial networks [59–61,64–66], the latter junior and mid-managerial leaders focus on planning, budgeting and coordination of the cooperation to carry out the mission within an established structure [59,61,66].

Two existing studies looked at facial cues to leadership attainment across different levels of management, but with contradictory findings. Re and Rule [67] found the perception of power predicts three levels of rank in law firms (including top-level managing partners) whereas perceived social-skill level predicts ranks in criminal organisations, for example, the Mafia. Re and Rule argue that since social skill is a quality shared by all lawyers, it should not differentiate lawyer rank. By contrast, the trait of power is thus a more distinctive quality that separates leaders from the followers of law firms. Contrary to this claim, in business organisations where the environment is similar to law firms, Linke [68] found facial trustworthiness was the sole predictor (non-significant predictors were facial attractiveness and dominance) of managers’ hierarchical positions. Both of these studies employed disproportionately few facial photos from the top-management level, yet claim a single perceived trait is predictive of success across career levels.

Building on these foundations, this paper seeks to explore the intersection of facial appearance and leadership perception among mid-level managers in a non-Western setting, with information supplied from Central China Real Estate Group. By examining how perceived leadership, apparent competence, trustworthiness, attractiveness and age influence leadership outcomes within a two-dimensional and an expanded three-dimensional social evaluation framework, this study aims to provide insights into how facial traits influence leadership attainment. This study exclusively looked at male leadership emergence, as the aim was to explore whether the results align with previous findings that also focused solely on male data. Addressing gender differences is a complex task and will be the focus of future studies. By initially confirming the findings within a male-only sample, we can build a solid foundation for subsequent research that includes a balanced gender representation.

### Derivation of Hypotheses

We will first examine the visual differences between mid-level managers and employees with no managerial roles (followers) to verify the relationship between apparent traits perceived from the face and leadership position in corporate business.

***H***_1_: Mid-level managers (leaders) will be rated differently in a set of facially perceived traits compared to employees without a managerial position (followers).

The power and warmth facial dimensions proposed by Todorov [41] are interpretations of two abstract factors comprised of distinct clusters of attributions. Competence and trustworthiness are used as approximations of the power and warmth dimensions in facial research [1,4,7,41,69]. We, therefore, expect that leadership positions can be predicted from ratings of perceived leadership, competence and trustworthiness.

***H***_***2***_***a:*** Actual leadership status will be predicted by ratings of perceived competence.

***H***_***2***_***b:*** Actual leadership status will be predicted by ratings of perceived trustworthiness.

***H***_***2***_***c:*** Actual leadership status will be predicted by ratings of perceived leadership.

Besides competence and trustworthiness, we will also explore the influence of perceived age and attractiveness on actual leadership attainment and leadership perception. We expect perceived age and attractiveness to have a positive effect on leadership perception and actual leadership attainment.

***H***_***3***_***a:*** Older faces will be perceived as more leader-like and age will be positively associated with actual leadership attainment.

***H***_***3***_***b:*** More attractive faces will be perceived as more leader-like and attractiveness will be positively associated with actual leadership attainment.

## Method

### Materials

A batch of male staff photos were supplied by Central China Real Estate Limited, which is one of the leading real estate firms in China. Images were exported from the company’s personnel management system by human resource officers. The management status of each depicted staff (i.e., whether the depicted person was a leader – has the responsibility to distribute and manage human and physical resources but excluding chief leadership positions, or a follower – no managerial responsibilities) was disclosed to researchers. All other information was omitted to protect staff privacy. Most of the photos were taken by the company’s photographer and followed the same standard: front view portrait against a light blue background. Almost all employees wore dark suits with formal shirts.

**Fig 1:**
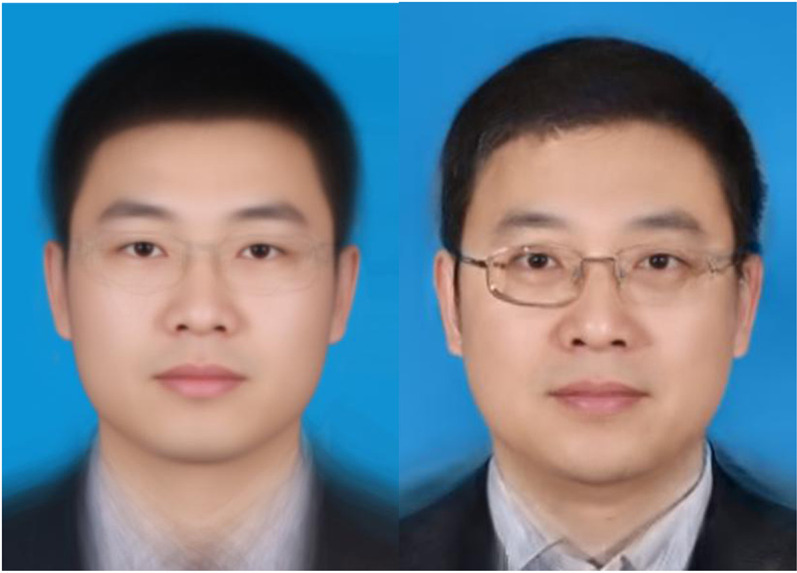
The average composite images of male follower and leader. The average male follower (from 50 follower faces) is on the left, the male leader (from 22 leader faces) is on the right.

Seventy-two male headshot photographs (N = 72, 22 leaders and 50 followers) were processed in this study. The images were standardised on pupil positions and cropped closely to the sides of the face. To visualise the differences in the facial features between leaders and followers, average composite images of leaders and followers were created respectively (see Fig 1) using the facial morphing software, Psychomorph [70]. To achieve this, 189 landmarks were placed on each face to serve as the basis of both structure and texture information. The average location of each point of the component faces was then calculated, and the individual face images were then warped into the relevant average shape and blended together to produce an average face. This technique captures the common facial characteristics shared by the faces entered into the morphing process.

### Participants and procedures

Five different sets of participants were recruited from the US via Amazon Mechanical Turk (MTurk) for ratings of competence (*N* = 31, 12 female, Mean (*M*_age_ = 30.87), trustworthiness (*N* = 32, 13 female, *M*_age_ = 36.52), attractiveness (*N* = 34, 13 female, *M*_age_ = 33.26), leadership (*N* = 108, 45 female, *M*_age_ = 37.44), and perceived age (*N* = 34, 11 female, *M*_age_ = 35.29). MTurk was chosen for its ability to recruit a large, diverse sample of participants efficiently, which enhances the generalisability and reliability of the findings [71,72]. Previous research has indicated that a sample size of around 20–50 participants per trait is sufficient to form reliable judgements of faces [2,73] in cross-racial perception studies [25,74]. This random sampling strategy is effective as it allows access to a broad demographic that is representative of the general population, ensuring robust and valid data for the study [75].

The studies were conducted online between 12 December and 31 December 2015. Participants in each task judged the 72 male faces for one of the given traits. Facial images within were shown in random order to participants. The procedures for the judgements of competence, trustworthiness, attractiveness, age and leadership were similar. Before starting the experiment, participants were informed the study was about “implicit perception towards faces”, and consent for using their ratings for research was collected. The methods employed were approved by the University of St Andrews Teaching and Research Ethics committee (PS11812).

Participants first completed a simple questionnaire regarding their background (ethnicity and country of residency). Then they were presented with one face at a time and instructed to rate “How competent/trustworthy/ attractive does this person look to you?” on a 7-point Likert scale, with one being “not at all” and seven being “very competent/ trustworthy/ attractive”. For the rating of leadership, the instruction reads “How good a leader do you think this person is?” A 7-point-scale was also used with one being “not at all” and seven being “very good”. For the judgement of perceived age, participants were asked: “How old does this person look to you?”. A slider was used for scaling the age of the face depicted, with 10 on the far-left end, and 80 on the far-right end. By dragging a coloured square along the slider, a number would appear underneath the photo, clearly indicating the numerical age the participant had chosen. All other procedures were identical to the other tasks.

## Results

### Preliminary analysis

Cronbach’s alpha values showed high-rater agreement for attractiveness (0.97), trustworthiness (0.96), competence (0.97), leadership (0.96) and age (0.98). The average rating across participants for each staff member photograph on personal traits (attractiveness, competence, trustworthiness, leadership and perceived age) was then calculated.

Average or composite images of leaders and followers were created (see Fig 1) to represent visually the facial features associated with leaders and followers, respectively. Apart from an evident age difference (the follower average appeared to look younger), the average leader was also wearing a pair of glasses while the average follower was not. Though almost half of the faces were wearing glasses (33 faces out of 72), 72.7% of leaders were wearing glasses compared to only 34.0% of non-leaders.

The average of each of the 5 ratings across the participants was calculated and average ratings were then intercorrelated. Perceived traits from the faces were interrelated (see details in Table 1). Pearson’s correlation showed perceived age had a weak positive relationship with leadership (*r* [72] =.32, *p* < .01) and competence (*r* [72] =.29, *p* < .05) and a weak negative relationship with attractiveness (*r* [72] = -.38, *p* < .001). Besides age, perceived leadership had strong positive correlations with competence (*r* [72] =.83, *p* < .001), trustworthiness (*r* [72] =.65, *p* < .001) and attractiveness (*r* [72] =.60, *p* < .001). Attractiveness positively correlated with competence (*r* [72] =.60, *p* < .001) and trustworthiness (*r* [72] =.56, *p* < .001). To our surprise, competence also moderately correlated with trustworthiness (*r* [72] =.55, *p* < .001). No correlation was expected between competence and trustworthiness as they are representative traits of the two orthogonal social dimensions used to evaluate faces.

**Table 1:** Summary of correlations among perceived traits.

	Age	Competence	Trustworthiness	Attractiveness
Leadership	.32^**^	.83^***^	.65^***^	.60^***^
Age		.29^*^	-.09	-.38^***^
Competence			.55^***^	.62^***^
Trustworthiness				.56^***^

Note: *N* = 72,

* *p* < .05,

** *p* < .01,

*** *p* < .001.

### Differences between leaders and followers

**Fig 2:**
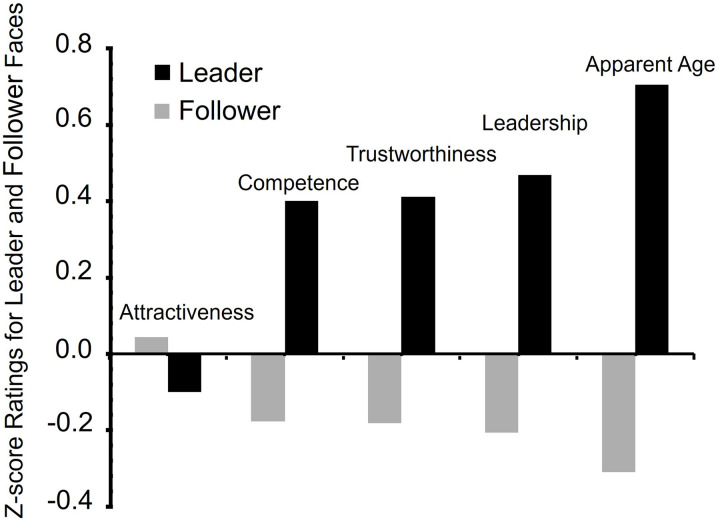
The Z-score mean ratings for leaders and followers. Ratings of attractiveness, competence, trustworthiness, leadership and age for actual male leaders (dark bars) and followers (light bars). Z scores applied across all variables to ensure uniform comparison in the bar graph.

Independent sample *t* tests were carried out to compare all ratings inferred from the face (perceived leadership, competence, trustworthiness, attractiveness and age) between leaders and followers. Despite being equally attractive (*t* = -.56 *M*_difference_ = .08, *p* = .58, *SE* = .15, 95% CI [-.37, .21]), the faces of the leaders received significantly higher average leadership ratings compared to the faces of the followers (*t* = 2.76, *M*_difference_ = .42, *p* = .007, *SE* = .15, *d* = .71, 95% CI [.12,.72]). Leader faces were also rated as older (*t* = 4.47, *M*_difference_ = 7.42 years, *p* < .001, *SE* = 1.66, *d* = 1.14, 95% CI [4.11, 10.73]), more competent (*t* = 2.35, *M*_difference_ = .33, *p* = .022, *SE* = .14, *d* = .60, 95% CI [.05,.62]) and *t*rustworthy (*t* = 2.40, *M*_difference_ = .28, *p* = .019, *SE* = .12, *d* = .61, 95% CI [.05,.52]) *t*han the faces of followers (see Table 2 and Fig 2, Z scores were utilised in Fig 2 to standardise the representation of perceived age—measured on an actual age scale—and other variables captured on a 7-point Likert scale, ensuring a uniform visual comparison). Follower faces were rated (non-significantly) more attractive than leader faces.

**Table 2:** Independent t-test between the ratings for leaders and followers.

Trait	*M*_leader_	*M*_follower_	*t*	Mean difference	S. E.	*d*
Leadership	4.24	3.82	2.76	.42^**^	.15	.71
Perceived Age	45.52	38.11	4.47	7.42^***^	1.66	1.14
Competence	4.75	4.42	2.35	.33^*^	.14	.60
Trustworthiness	4.13	3.84	2.40	.28^*^	.12	.61
Attractiveness	3.27	3.35	-.56	-.08	.15	.14

Note: *df* = 70, *d* is Cohen’s d,

* *p* < .05,

** *p* < .01,

*** *p* < .001.

### Actual leadership attainment prediction

#### Addressing multicollinearity and class imbalance in the analysis.

To identify whether naive judgements made from the face alone predicted actual leadership status (leader or follower), we built several binary logistic regression (LR) models using perceived leadership, competence, trustworthiness and attractiveness as predictors. Preliminary analysis showed perceived leadership highly correlated with perceived competence, trustworthiness and attractiveness (see Table 1). To avoid possible collinearity, we built regression models with perceived leadership as the predictor for each of the other perceived ratings (competence, trustworthiness and attractiveness) and saved the residuals for each rating. These residuals (residual competence, residual trustworthiness, residual attractiveness) were then used as predictors in the hierarchical LR model. Perceived age was directly incorporated without adjustments, as its correlation with leadership and other perceived ratings was modest, and variance inflation factor (VIF) assessments with the residuals indicated no multicollinearity concerns (VIF for age: 3.45, attractiveness residual: 3.05, other ratings <= 1.35). Wearing glasses was also included in the model to test it as a possible confounding factor. The Box-Tidwell test was performed to ensure the linearity assumptions were met for all perceived ratings (leadership, age, competence, trustworthiness and attractiveness). No outliers were identified by examining Cook’s influence statistics (larger than 1), leverage value (between 0 and 1) and residuals (absolute value larger than 3).

In addressing the imbalanced dataset with leaders constituting a minority (22 out of 72 faces) and followers predictably dominating correct classifications in the non-leader category, we adjusted classification cut-off threshold in the logistic regression from chance level 0.5 to 0.406, informed by both the Youden’s Index and the maximum Kolmogorov-Smirnov statistic, and adjusted case weighting to 1.44 for followers and 3.27 for leaders. This strategy enhanced the balance between classifying true leaders and followers, achieving a high Receiver Operator Curve (ROC) analysis, Area Under the Curve (AUC = .871), and the best F1 score (an indicator of balance between recall and precision, which is a measure of a test’s accuracy in binary classification) of approximately 0.85, indicating a robust model with high discriminative power as corroborated by the ROC analysis.

#### Hierarchical Binary Logistic Regression analysis.

In the first logistic regression model (LR Model 1), perceived leadership was the sole predictor for actual leadership status (leader or follower). The test of LR Model 1 against the constant was significant (*χ*^2^ = 18.22, *p* < .001, *df* = 1, Nagelkerke’s R^2^ = .16, 64.6% correct prediction, F1 score = 0.687) which means the odds of a face being a leader are 3.584 times as high for each one-unit increase in perceived leadership (*B*_leadership_ = 1.276, *p* < .001, odds ratio = 3.584).

Perceived competence and trustworthiness residuals were added to LR Model 2, and this model was significant when compared against the constant (*χ*^2^ = 19.74, *p* < .001, *df* = 3, Nagelkerke’s R^2^ = .17, 61.6% correct prediction, F1 score = 0.671), though it was not significantly different from LR Model 1 (Δ*χ*^2^ = 1.51, *p* = .469, *df* = 2) which means this model of perceived leadership together with perceived competence and trustworthiness residuals did not predict actual leadership status better than perceived leadership alone (*B*_leadership_ = .1.29, *p* < .001, odds ratio = 3.621). Neither perceived competence residuals (*B*_competence_ = -.00, *p* = .984, odds ratio = .997) nor trustworthiness residuals (*B*_trustworthiness_ = .23, *p* = .222, odds ratio = 1.262) were significant predictors.

LR Model 3 added the perceived attractiveness residuals and perceived age to the predictors. LR Model 3 was significant when compared against the constant (*χ*^2^ = 65.99, *p* < .001, *df* = 5, Nagelkerke’s R^2^ = .49, 74.9% correct prediction, F1 score = 0.767) and was also a significant improvement over LR Model 2 (Δ*χ*^2^ = 46.25, *p* < .001, *df* = 2). Perceived trustworthiness residuals (*B*_trustworthiness_ = 1.00, *p* < .001, odds ratio = 2.718) and age (*B*_age_ = .23, *p* < .001, odds ratio = 1.258) became significant predictors in the model. Perceived leadership remained a significant predictor (*B*_leadership_ = 1.11, *p* = .043, odds ratio = 2.272). Attractiveness (*B*_attractiveness_ = -.104, *p* = .803, odds ratio = .901) was not a significant predictor.

Finally, LR Model 4 introduced the categorical variable of wearing glasses. LR Model 4 was significant compared against the constant (*χ*^2^ = 71.66, *p* < .001, *df* = 6, Nagelkerke’s R^2^ = .52, 84.4% correct prediction, F1 score = .849) and against LR Model 3 (Δ*χ*^2^ = 5.67, *p* = .017, *df* = 1). It is the most robust model across all four models. Perceived trustworthiness residuals (*B*_trustworthiness_ = 1.14, *p* < .001, odds ratio = 3.117), erceived age (*B*_age_ = .27, *p *< .001, odds ratio = 1.314) and glass-wearing (*B*_glasses_wearing_ = 1.333, *p* = .018, odds ratio = 3.792) were the significant predictors. When a face was rated higher on trustworthiness by one point, that face was 311.7% more likely to belong to an actual leader; similarly, for each additional year in perceived age, the face was 31.4% more likely to belong to an actual leader. If the person was wearing glasses, the odds of being perceived as a leader were 379.2% higher compared to those not wearing glasses (see Table 3).

**Table 3:** Binary logistic regression models predicting actual leadership.

	LR Model 1		LR Model 2		LR Model 3		LR Model 4	
Included	*b*	Exp(*B*)	*B*	Exp(*B*)	*b*	Exp(*B*)	*b*	Exp(*B*)
Constant	-5.152	.006	-5.204	.005	-12.993	.000	-13.407	.000
Perceived Leadership	1.276	3.584	1.287	3.621	.821	2.272	.307	1.360
Perceived Competence (residuals)			-.003	.997	-.072	.930	-.309	.734
Perceived Trustworthiness (residuals)			.232	1.262	1.000	2.718	1.137	3.117
Perceived Attractiveness (residuals)					-.104	.901	.492	1.636
Perceived Age					.230	1.258	.273	1.314
Glasses (category)							1.333	3.792
χ2(df)	18.223(1)^***^		19.736(3)^***^		65.988(5)^***^		71.661(6)^***^	
Step χ2(df)	18.223(1)^***^		1.513(2)		46.252(2)^***^		5.673(1)^*^	
Nagelkerke r^2^	.159		.171		.490		.523	
F1 Score	0.687		0.671		0.767		0.849	
Hosmer and Lemeshow *p*	<.001		.022		.108		.581	
Overall predicting rate	64.6%		61.6%		74.9%		84.4%	

Note:

* p < .05,

** p < .01,

*** p < .001. Residuals refer to variation in a perceived trait after controlling for variance, reflecting the trait’s relationship with perceived leadership.

#### Final Binary Logistic Regression analysis.

In an effort to refine the predictive model, a final binary logistic regression was conducted utilising only the significant predictors from previous analysis in the best model. This logistic regression included perceived trustworthiness (not residuals), age, and glass-wearing as predictors—with 0.306 adjusted for the classification threshold and no case weight adjustments. This decision was influenced by exploring the weighted model’s Hosmer and Lemeshow test. This model achieved a strong overall prediction rate of 81.9%.

The results of this logistic regression (*χ*2 = 27.71, *p* < .001, *df* = 3) suggested that both perceived trustworthiness (*B*_trustworthiness_ = 2.136, p = .012, odds ratio = 8.463) and age (*B*_age_ = .190, *p* = .001, odds ratio = 1.209) remained strong significant predictors of leadership status. Wearing glasses did not retain its significance in this model (*B*_glasses_wearing_ = .563, p = .410, odds ratio = 1.756). Wearing glasses was still not significant (*B*_glasses_wearing_ = .763, p = .086, odds ratio = 2.144) with the same weight (1.44 for follower and 3.27 for leader) and threshold setting (0.406). This shift could suggest that perceived trustworthiness and age are more robust predictors of leadership status than the temporary adornment of glasses.

## Discussion

This study contributes significantly to the cross-cultural validation of the social evaluation framework by demonstrating that perceived trustworthiness and age are paramount in predicting actual leadership status within a Chinese business cooperation context. Managers were seen as older, more competent, more trustworthy and better leaders but not more attractive than their colleagues who did not have management duties. These findings confirm that leadership attainment in a corporate setting in China prioritises trustworthiness and maturity over conventional attractiveness. The study enriches our understanding of leadership perception by integrating the third dimension of attractiveness/youth, as proposed by Sutherland [2], alongside the competence (power) and trustworthiness (warmth) model.

Our results underscore the complex interplay between facial cues and leadership perception and emergence, revealing that perceived age and trustworthiness supersede other predictors, including competence, attractiveness and general leadership appearance. This challenges the traditional emphasis on competence as a universal predictor of leadership, suggesting that trustworthiness and age are more critical in the context of mid-level management. In line with the results of Linke [68] within a Western context, perceived trustworthiness was found here to be a major predictor of leadership success in a Chinese business cooperation setting. This finding confirms a relationship between attributions made from facial appearance and actual leadership success for middle level management, even when leaders are chosen not by bottom-up election but from top-down appointment following promotion procedures that usually require clear evidence of competency.

Though trustworthy is the term we used in our ratings, we should not forget facial trustworthiness approximates the valence dimension which signals approachability and positive emotion. There is an apparently more positive emotion in the leader’s average image compared to the follower’s average (see Fig 1). The perceived differences in trustworthiness and the association between trustworthiness and actual leadership position may be driven by the facial cues to warmth or emotional positivity.

Perceived competence did not contribute to actual leadership status as good as perceived trustworthiness. This unexpected finding underscores the role of cultural context in shaping leadership perceptions. Chinese organisations are known for their interdependent culture [76–79], which is rooted in Confucianism [35] that celebrates the core value of interpersonal harmony and seniority based on social hierarchy epitomised by the Senpai–Kouhai relationship (where junior, inexperienced Kouhai show respect for older, more senior Sempai in social interactions) [80]. Everyone is woven into a tightly connected social network [76,77,79,81]. As the middle and lower managers are often appointed by their superordinate, the approachability and ability to quickly gain the trust of others may be more advantageous for the individual to exert influence in the social web, thus providing value to their superordinate. On the other hand, apparent competence may make one stand out from the crowd. It might attract unwanted attention and trigger concerns of rivalry. Therefore, apparent trustworthiness may matter more than apparent competence in climbing the lower-level organisational hierarchy.

As we focus on the comparison of the middle level of managers and employees, the prediction of actual leadership by age is somewhat expected. Typically, age coincides with experience which is a critical component in career advancements outside the executive boardroom. Leadership traits and behaviours that are commonly seen at this management level may be drastically different from the traits required in the top executive level. It is a common assumption that requirements for progression on the career ladder are linear: that is to assume continually enhancing personal factors which have facilitated leadership attainment at a lower level will also benefit achievement of progressively higher leadership positions. In reality, many employees experience a block in career advancement suggesting there are distinctive personal factors required for high-level management, and these distinctive characteristics may be rare. The mean score on the competence of the middle and lower-level leaders in our data is 4.75 out of 7. Hence, most of the managers in our studies did not look exceptionally high in competence. In Linke et al.’s [68] study where they found trustworthiness to be the sole predictor of leadership position, despite sampling across several different organisations, a disproportionately small number of leaders were at the top rank. It is possible that both our study and that of Linke [68] did not have a large enough sample at the higher level to find the effect of apparent dominance on promotion. That is to say, apparent competence could be a scarce facial feature not commonly possessed outside elite leaders and hence not presented in our photo set. Our data, therefore, do not contradict the findings of Rule and Ambady [82] or Re and Rule [67] that competence and other power-related traits predict leadership ranks because the face sets they studied include business CEOs and elite law practitioners.

An interesting effect of glasses-wearing on leadership perception was found in this study. Glasses as one common facial ornament are usually excluded from analysis to control undesired noise, yet wearing glasses is extremely common among today’s workforce. In our study, 75% leaders vs 34% followers wore glasses. This difference may reflect a difference in style or preference where leaders are more comfortable, and likely to choose wearing glasses, compared to employees during photography, but we do not have data on style or preferences to make further analysis.

### Limitations and future directions

There are several possible limitations of the current study. Perceived age (maturity) is known to be highly correlated with perceived dominance/power up to 35 years old for male faces [54]. In this study, the mean perceived age of leaders was 45.5 and the mean perceived age of the followers was 38.1 which are both above 35. Therefore, the effect of perceived age on leadership attainment found in our data is unlikely to be driven by perceived dominance. Ratings on dominance, power, likeability, and approachability, could be collected to explore and compare different models of power-valence aggregated ratings on leadership perception and attainment.

Social judgements from faces predicted actual management positions but it is too soon to conclude the direction of cause and effect, although it is that likely trustworthy-looking individuals are given more opportunities to be selected as leaders. We cannot eliminate the possibility that people with management status enjoy better pay and lifestyle which in turn enables them to display and perhaps feel positive emotions more easily.

Future research should further explore the effect of culture in leadership perception, employing a more diverse range of participants and facial images, especially with female leaders and followers to dissect the cultural specificity and universality of leadership traits. Additionally, expanding the array of character ratings could unveil more detailed insights into the conditional nature of leadership perception.

## Conclusion

Our findings illuminate the critical roles of perceived trustworthiness and age in leadership perception and attainment, advancing the facial social evaluation framework’s cross-cultural applicability. This study demonstrates the impact of facial appearances on managerial status attainment in a business cooperation in a global context. These data add to the evidence of the link between physical appearance and leadership emergence, contributing valuable insights to the fields of perception and leadership studies.

## Supporting information

S1 File Addressing class imbalance in logistic regression. (PDF)

S1 Fig RoC curves for models using weighted cases with informed classification cut off at .406. (TIF)
