# Supplementary material for: Validation of facial attributions in leadership: Trustworthiness and age in Chinese mid-level management
Source: PLoS One. 2025 May 27;20(5):e0324508. doi: 10.1371/journal.pone.0324508 (PMC12111254; doi:10.1371/journal.pone.0324508)
Supplement: S1 File — (PDF) [file pone.0324508.s001.pdf]

Validation of facial attributions in leadership: Trustworthiness and age in Chinese mid-level management

Jing Rachel Ma[1*, 2], David Ian Perrett[1¶]

1 School of Psychology & Neuroscience, University of St. Andrews

2 School of Business, University of Dundee

*Corresponding author information:

Email: jma001@dundee.ac.uk (JM)

Supplement 1: addressing class imbalance in logistic regression

To mitigate potential bias introduced by class imbalance (22 leaders vs. 50 followers), we explored different strategies including resampling, SMOTE (Synthetic Minority Over-sampling Technique), ridge regression, threshold adjustment [1], classification cut-off setting in logistic regression model[2], and adjust case weights [3]. We systematically evaluated different techniques to improve classification performance. The goal was to ensure that **our statistical model fairly represents both groups**, balancing sensitivity and specificity while maintaining predictive accuracy.

We considered and evaluated the following approaches, and the first two approaches were disregarded:

1. Resampling-based methods (e.g., SMOTE, undersampling, oversampling)
   - These techniques were ruled out due to the relatively small dataset size, which could lead to overfitting and artificial inflation of sample variance.
2. Ridge Regression (L2 Regularization)
   - Disregarded as it primarily reduces overfitting rather than correcting class imbalances.
3. **Threshold Adjustment & Case Weighting**, two primary strategies were selected:
   - **Classification Cutoff Adjustment:** Changing the probability threshold at which a case is classified as a leader. Probability for each case were saved from each analysis to use for ROC curve analysis to evaluate the discriminatory ability of model and calculate AUC values.
   - **Case Weighting:** Assigning different weights to leader and follower cases to balance their impact in the model.

Model Comparison & Selection of Optimal Approach

We implemented logistic regression under four conditions and evaluated their performance using Precision, Recall, F1 Score, ROC Curve, and AUC metrics (see S1 Table 1).

The classification threshold was initially set to opportunity level at 0.5 (Mode 0), then proportional to the minority class -the leaders' frequency at 0.306 (Mode 1). Additionally, case weights were experimentally determined to balance the influence of both classes in the model, with the minority cases of the leaders adjust to 3.27 and followers adjust to 1.44 at chance level initially (Mode 2). We then examine and compare these models on key indicators including recall and precision, F1 score, RUC curve and AUC metrics (see details in S1 Table 1), and Mode 2 - the weighted case model with a 0.5 cutoff emerges as the most balanced approach, achieving the highest recall and F1 score. It already effectively identifies a larger proportion of true leaders without a significant compromise in precision. The Max K-S is high at 0.689 with a suggested cut-off at 0.406. it is the optimal threshold that distinguish between followers and leaders with the highest balance between sensitivity and specificity. Thus, we adjust the classification cut-off value to 0.406 with weighted cases, and this time achieved the highest F1 score of approximately 0.85 which indicates a very good balance between recall and precision, indicating a robust model with high discriminative power as corroborated by the ROC analysis (AUC = 0.871) (see S1 Fig 1).

Calculation of proportion of leaders:

22 leaders out of 72 total faces, the proportion of leaders is $22/72 \approx 0.306$ (follower frequency is at 69.4% and leader is at 30.6%,)

Calculation of Weights:

$Weight_{leader} = 1 / 0.306 \approx 3.27$

$Weight_{follower} = 1 / 0.694 \approx 1.44$

Calculation of Precision, Recall, F1 Score:

Recall = TruePositives / (TP + FalseNegatives)

Precision = TP / (TP + FalsePositives)

F1 Score = 2 * (Precision * Recall) / (Precision + Recall)

S1 Table1 Comparison of Logistic Regression Models with Different Classification Cutoffs.

Model Description	*Cutoff Value*	*Precision*	*Recall*	*F1 Score*	*AUC*
Mode 0 (no weight adjustment*)*	0.5	0.591	0.684	0.634	.872
Mode 1 (no weight adjustment)	0.306	0.818	0.643	0.719	.872
Mode 2 (Weighted cases)	0.5	0.778	0.800	0.789	.871
Mode 3 (Weighted cases)	0.406	0.802	0.903	0.850	.871

Note. this table presents the precision, recall, and F1 scores for four different logistic regression models. Mode 0 represents the baseline model with no weight adjustments and opportunity classification cut-off at .5, Mode 1 applies a simple classification cutoff set at the proportion of the minority class - leaders, Mode 2 uses weighted cases with a standard 0.5 cutoff, and Mode 3 employs weighted cases with a cutoff informed by the Kolmogorov-Smirnov statistic. AUC values are calculated from ROC analysis for evaluating the discriminatory ability of model.

S1 Fig1. ROC Curves for Models Using weighted cases with informed classification cut off at .406

References

1. Fawcett T. An introduction to ROC analysis. Pattern Recognit Lett. 2006;27: 861–874. doi:10.1016/j.patrec.2005.10.010

2. Kleinbaum DG, Klein M. Logistic Regression. New York, NY: Springer New York; 2010. doi:10.1007/978-1-4419-1742-3

3. King G, Zeng L. Logistic Regression in Rare Events Data. Polit Anal. 2001;9: 137–163. doi:10.1093/oxfordjournals.pan.a004868
