# Supplementary figures and images for: Validation of facial attributions in leadership: Trustworthiness and age in Chinese mid-level management
Source: PLoS One. 2025 May 27;20(5):e0324508. doi: 10.1371/journal.pone.0324508 (PMC12111254; doi:10.1371/journal.pone.0324508)

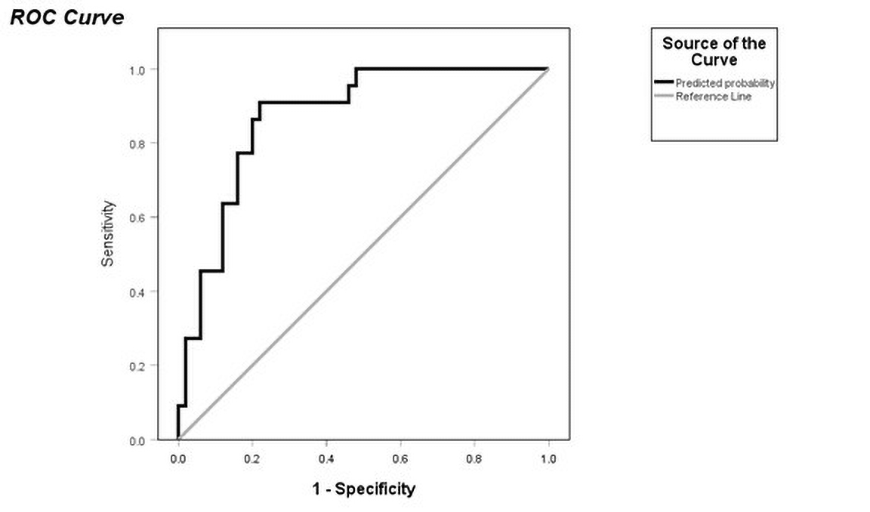

Supplement: S1 Fig — (TIF) [file pone.0324508.s002.tif]
